# Prevalence of scabies and bacterial skin infection in French Polynesia: A cross-sectional community survey

**DOI:** 10.1371/journal.pntd.0013119

**Published:** 2025-06-09

**Authors:** Georgia R. Walker, Susanna J. Lake, Matthew G. Parnaby, Tereva Reneteaud, Mihiau Mapotoeke, Romain P. Marmorat, Raihei H. White, Sarah Andersson, John M. Kaldor, Jean-Marc Segalin, Henri-Pierre Mallet, Andrew C. Steer, Andre L. Wattiaux

**Affiliations:** 1 Murdoch Children’s Research Institute, Melbourne, Australia; 2 Office of Health Surveillance and Response, Agence de Régulation de l’Action Sanitaire et Sociale (Regulatory Agency for Health and Social Action), Papeete, French Polynesia; 3 Kirby Institute, University of New South Wales, Sydney, Australia; 4 Rheumatic Heart Disease (RHD) Centre, Department of Health, Pirae, French Polynesia; Hebrew University Hadassah Medical School, ISRAEL

## Abstract

**Introduction:**

The prevalence of scabies and impetigo is high in several Pacific Island countries and has led to the implementation of mass drug administration control strategies. Anecdotal clinical reports have suggested similarly high burdens of scabies and bacterial skin infection in French Polynesia, but formal prevalence data have been lacking. We aimed to determine the prevalence of scabies and bacterial skin infection in French Polynesia’s two most populated islands: Tahiti and Mo’orea.

**Methods:**

We conducted a community-based cross-sectional survey. Our study was pragmatically restricted to the main islands of Tahiti and Mo’orea, where we selected households using two-stage randomisation from 20 first-stage neighbourhoods. All individuals present in the home were invited to participate. Four nurses and one doctor conducted skin examinations using validated simplified diagnostic criteria to diagnose scabies, informed by the International Alliance for the Control of Scabies 2020 diagnostic criteria.

**Results:**

Among 1770 participants, scabies prevalence was 11.9% and was highest among children aged <5 years (31.8%, RR 5.7, 95% CI 3.8 – 8.6, compared to ≥55 years). The overall bacterial skin infection prevalence was 5.5%. Participants with scabies had a 10-fold higher risk of bacterial skin infection compared to those without (26.8% vs 2.7%, RR 10.2, 95% CI 7.5–13.8). Written consent was obtained for all 1770 participants.

**Conclusions:**

The prevalence of scabies in this study exceeds 10%, the threshold above which evidence-based public health strategies including mass drug administration (treatment of the entire community, regardless of whether they show evidence of the disease) may be considered.

## Author summary

Scabies is a skin infestation due to the mite *Sarcoptes scabei* var *homini* which causes intense itch, negative impact on quality of life, and commonly leads to secondary bacterial skin infections and their sequelae. Anecdotal reports suggest high burdens of scabies and bacterial skin infection in French Polynesia, but prevalence data have been missing. We aimed to quantify the prevalence of these skin diseases in the main islands of Tahiti and Mo’orea. In a cross-sectional survey, we used validated simplified diagnostic criteria to diagnose scabies, and found that 11.9% of 1770 participants had scabies, and 5.5% had bacterial skin infection, which is comparable to rates found in other Pacific contexts. Participants with scabies were ten times more likely to have bacterial skin infection, and children carried the highest burden of both diseases. These results support the need for community-wide public health interventions to reduce the prevalence of scabies, such as through mass drug administration.

## Introduction

Scabies is a parasitic skin infection and a neglected tropical disease (NTD) caused by the microscopic mite *Sarcoptes scabei* var *hominis*.[1] Infection is transmitted from human to human by close contact with the skin, and is more common in impoverished and overcrowded settings.[2] The direct effects of infestation include debilitating itch, and disrupted sleep, concentration, education and employment. Scratching can lead to breaches in the skin barrier and secondary bacterial infection, most frequently with Streptococcus pyogenes and Staphylococcus aureus. Bacterial skin infection can present as localised impetigo, infected scabies lesions or more complicated skin infection, and can lead to sepsis. Streptococcus pyogenes infections may contribute to chronic immune-mediated diseases of the heart and kidneys including rheumatic heart disease and post streptococcal glomerulonephritis.[3]

Increasingly, scabies is being recognised as a global public health priority. The World Health Organization (WHO) has published targets for control and elimination measures to be met by 2030, and has called for evaluation of the epidemiological burden of scabies.[1] Systematic reviews have shown population-based scabies prevalence ranging from <1 – 71%, though few of the studies have used comparable methodology.[4] Since 2007, a series of prevalence surveys conducted in Pacific Island countries has employed a semi-standardised methodology, confirming high prevalence of scabies (23.6% in Fiji, and 15.0% in Solomon Islands’ Western Province) and impetigo (19.6% and 5.6% respectively).[5,6] These results have provided an evidence-based rationale for scabies control strategies. Mass drug administration (MDA) with ivermectin has been shown to be highly effective in reducing prevalence of scabies and associated impetigo, and is recommended where community scabies prevalence is greater than 10%.[7,8]

Several NTDs affect the health and wellbeing of French Polynesian society. The country experiences periodic outbreaks of dengue, sporadic leprosy, and declining rates of lymphatic filariasis since an MDA elimination strategy was implemented in the early 2000s.[9–12] While anecdotal clinical reports are suggestive of a high burden of disease of scabies and bacterial skin infection in French Polynesia, there are no community-based published data to quantify prevalence. We undertook a prevalence survey of scabies and bacterial skin infection in the two most populous islands of French Polynesia to determine the burden of disease and demographic risk factors, with a view to inform future control strategies.

## Methods

### Ethics statement

The study was approved by the Royal Children’s Hospital Human Research Ethics Committee, Melbourne, Australia (Ref: 102043) and formally supported by the French Polynesian Government. All participants provided written consent to participate. A parent or guardian provided written consent for people aged less than 18 years and for people unable to provide their own consent, and mature minors also provided written assent.

### Setting

**Fig 1:**
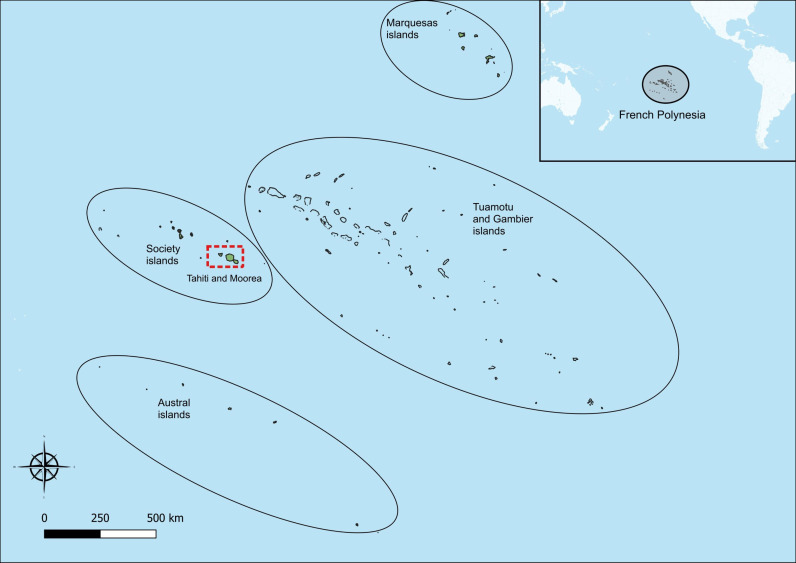
Study area in the islands of Tahiti and Mo’orea, French Polynesia. The source that was used to create this map is found at data.gouv.fr. The authors understand and agree to the terms of the Creative Commons Attribution 4.0 International (CC BY 4.0) License.

French Polynesia is a South Pacific Island country and an autonomous overseas collectivity of France, comprising five archipelagos with 121 islands and atolls (Fig 1). It spans a sea mass of 5.3 million square kilometres (approximately 70% the land mass of Australia) and has a population of 278,786 people (census data, August 2022).[13] French Polynesian society comprises a wide range of socioeconomic strata with universal health coverage and varying levels of access to private medical care. The training component of this study was completed at the Agence de Régulation de l’Action Sanitaire et Sociale (ARASS, the Regulatory Agency for Health and Social Action) in the capital city, Papeete, on the island of Tahiti.

We conducted a community-based, cross-sectional prevalence survey to determine the prevalence of scabies and bacterial skin infection in French Polynesia’s two most populated islands. The design was pragmatic, accounting for resource limitations and the associated infeasibility of sampling all of French Polynesia’s five archipelagos. The study was localised to the Îles du Vent (Windward Islands) of Tahiti and Mo’orea, where approximately 75% of the country’s population lives. Tahiti is the country’s most populated island, the site of its capital city, and the central hub for inter-island travel routes. French Polynesians living in outer islands travel to and from Tahiti for reasons of employment, education, socialisation and health, and to access other outer islands. Mo’orea is a smaller island located a 30 minute ferry ride from Tahiti. For these reasons, a prevalence survey conducted in Tahiti and Mo’orea was considered likely to yield findings relevant to other parts of the country.

### Sampling strategy

We conducted a single survey across the islands of Tahiti and Mo’orea. We ensured that there was representativity of urban and rural populations by first dividing the islands of Tahiti and Mo’orea into two evaluation units (EUs) of similar size, with EU 1 urban and EU 2 rural. EUs were based on existing administrative divisions and island geography. An EU is typically a population range of 100 000 – 250 000 and corresponds to one or multiple administrative units for health service provision, provided they can be meaningfully grouped.[14] Randomisation of first-stage clusters was then performed for each EU.

We used the World Scabies Program sampling strategy for baseline prevalence surveys to inform our sample size estimation.[14] To detect an estimated 20% scabies prevalence with 4% precision, and with a design effect of 2 for clustering, we estimated that a total of at least 1534 participants were required, i.e., at least 767 participants in each EU. We aimed at 2000 participants (i.e., 1000 in each EU) to enhance study power.

We used a two-stage random cluster sample with 20 first-stage clusters (10 from each EU) randomly selected from a comprehensive list of villages and lower urban administrative units (“neighbourhoods”) using SMART software.[15] Probability of selection was proportional to the population size of each village or neighbourhood (hereafter collectively “neighbourhoods”), thus providing an equal opportunity for individuals to be surveyed irrespective of the size of the setting they lived in. For second-stage clusters, we used an aerial map of each neighbourhood and randomly chose a starting point and direction of travel using a geographic information system software (QGIS 3.22.4). Field teams approached every third household on the same side of the street as the starting point aiming for 100 recruited participants per neighbourhood.

### Study procedures

We selected five local health professionals (four nurses and one doctor) to train in skin examination technique and to recognise scabies lesions and bacterial skin infection using validated, simplified criteria.[16] Five local staff (data officers, nurses and epidemiologists) were trained in data capture. Overall project supervision, including training in field procedures, was provided by GW and AW. All team members received training in research ethics, the study’s methodology, code of conduct and child safety. Training spanned two days and was conducted in French. Country-wide communications with health services and press releases preceded the recruitment phase. In each neighbourhood the field team visited the local publicly funded healthcare centre to advise health personnel that recruitment was taking place on that day, and that patients with features of skin infection or another incidental medical concern would be referred to their care.

Field teams were each comprised of one local health professional as the skin examiner, and one data officer. Between one to four teams were deployed each day for three weeks, depending on availability. Participant information sheets were provided in French and Tahitian. All individuals present in each household were invited to participate. Participants (or their parents or guardians) provided verbal and written consent and were then asked their age, sex, the number of people who usually resided in their home, whether they usually lived in French Polynesia, and whether they had experienced itching and/or scratching in the previous 24 hours.

Participants underwent a skin examination of the limbs from the elbows and knees down, and the field team recorded the presence and number (≤10 vs. > 10) of scabies lesions and signs of bacterial skin infection on hand-held digital devices. Mild scabies disease was classified as ≤10 lesions, with >10 lesions indicating moderate to severe disease.[6,17] Participants with signs of scabies, bacterial skin infections, or other incidentally noted health conditions, were provided a written referral to their local health service for formal diagnosis and treatment.

Scabies was defined as ‘typical lesions and distribution on exposed skin, with or without itch’, per simplified criteria.[16] These criteria were selected to allow for the rapid assessment of participants. Bacterial skin infection was defined as impetigo (papular, pustular or ulcerative lesions surrounded by erythema, with or without crusts, pus or bullae), infected scabies, boils, abscesses, and cellulitis.

### Statistical analysis

We compared demographic characteristics of age, sex, commune, urban/rural, usual household occupancy and resident status to census data from 2017 (the most recent completely published dataset). We calculated prevalence of scabies and bacterial skin infection for the whole sample and across demographic categories using those aged more than 54 years as the reference group for calculating relative risk. A Poisson regression model was used to model scabies and bacterial skin infection, adjusted for clustering by neighbourhood. Quantile regression with clustering by neighbourhood was used to compare medians of household occupancy. We calculated cluster-adjusted population attributable fraction ([incidence in total population – incidence in unexposed population]/incidence in total population) as a measure of the impact of scabies on the population prevalence of bacterial skin infection. X^2^ tests were used for categorical data and t-tests for continuous data. Statistical output included relative risk with 95% confidence interval, and p values of <0.05 were considered statistically significant. All statistical analyses were conducted using Stata statistical software version 18.0 (Stata Corp., College Station, United States of America).

## Results

### Demographic characteristics

We recruited 1770 participants across 20 neighbourhoods in 13 communes during the period 6^th^ to 23^rd^ November 2023. Enrolment ranged from 64 to 105 participants per neighbourhood. Demographic characteristics of the study cohort were generally reflective of the overall French Polynesian population characteristics based on census data (Table 1). More females and people aged over 54 years participated in the study compared to the census (58.3 vs. 49.5%, and 27.7% vs. 19.9%, respectively).

**Table 1:** Demographic characteristics of the study sample compared to 2017 national census data [18].

		Study (n = 1770)		Census n = 207 333
Characteristic (n)		n	% (95% CI)	%
Gender (n = 1767)	Female	1030	58.3 (55.8-60.1)	49.5
	Male	737	41.7 (39.3-44.2)	50.5
Age (years) (n = 1761)	<5	129	7.3 (6.0-8.9)	6.4
	5-9	118	6.7 (5.2-8.7)	7.8
	10-14	83	4.7 (3.4-6.6)	7.8
	15-24	227	12.9 (10.9-15.2)	15.6
	25-34	240	13.6 (11.8-15.7)	16.1
	35-54	476	27.0 (25.4-28.7)	28.7
	≥55	488	27.7 (25.1-30.5)	17.7
Usually residing in French Polynesia (n = 1764)	Yes	1700	96.4 (94.6-97.6)	Not available
Location	Urban	911	51.5 (28.7-73.6)	(108 986) 52.6
	Rural	859	48.5 (26.4-71.3)	(98 347) 47.4

### Household recruitment

Our 1770 participants were recruited from 503 households (average household recruitment of 3.5 participants). The reported usual household occupancy (“how many people usually live in this home?”) ranged from one to 30 people (median 5, IQR 3–7). In 16 of the 20 neighbourhoods the team documented success or failure of recruitment by household approached. Recruitment failure occurred when a house was unoccupied or abandoned, or if its occupants declined to participate. Within these 16 neighbourhoods, the team successfully recruited participants from 356 out of 740 (48%) households approached.

### Scabies

**Fig 2:**
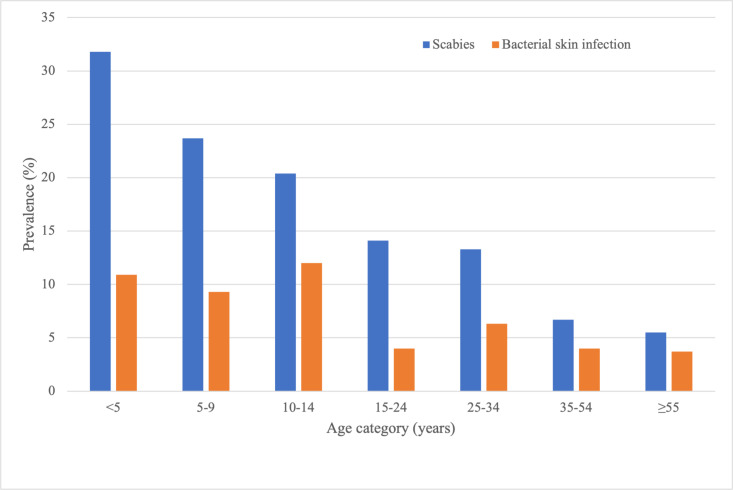
Prevalence of scabies and bacterial skin infection by age category.

We observed scabies among 210 participants (11.9%, 95% CI 9.7 - 14.5%, Table 2). Prevalence was highest among children aged less than 5 years (31.8%, RR 5.7, 95% CI 3.8 – 8.6, compared to those aged over 54 years, Fig 2). The prevalence of scabies was similar in urban and rural areas. Of the 210 participants with scabies, 57 had at least 10 scabies lesions (27.1%, 95% CI 20.7 – 34.7%). There were no cases of crusted scabies. A history of itch or scratching in the 24 hours preceding skin assessment was reported by 187 participants (10.6%, 95% CI 8.7 – 12.8%), and was more common among those with scabies than those without (47.1% vs. 5.7%, RR 8.3, 95% CI 6.2–11.1). Household occupancy was higher among participants with scabies than those without (median 6 inhabitants [IQR4–7] vs. median 5 inhabitants [IQR3–7], P = 0.016).

**Table 2:** Prevalence of scabies and bacterial skin infection in French Polynesia.

		Scabies (n = 1770)			Bacterial skin infection (n = 1769)		
		n/N	% (95%CI)	Relative risk (95%CI)	n/N	% (95%CI)	Relative risk (95%CI)
Total		210/1770	11.9 (9.7-14.5)		97/1769	5.5 (4.1-7.4)	
Gender	Female	121/1030	11.7 (9.2-14.9)	Ref	50/1030	4.9 (3.4-6.9)	Ref
	Male	89/737	12.1 (9.4-15.4)	1.0 (0.8 – 1.3)	47/736	6.4 (4.7-8.6)	1.3 (0.99 – 1.7)
Age (years)	<5	41/129	31.8 (21.9-43.6)	5.7 (3.8 – 8.6)	14/129	10.9 (6.7-17.1)	2.9 (1.6 – 5.4)
	5-9	28/118	23.7 (15.3-34.9)	4.3 (2.4 – 7.6)	11/118	9.3 (5.0-16.7)	2.5 (1.4 – 4.5)
	10-14	17/83	20.4 (11.6-33.6)	3.7 (2.1 – 6.4)	10/83	12.0 (6.6-21.1)	3.3 (2.0 – 5.4)
	15-24	32/227	14.1 (8.8-21.6)	2.5 (1.5 – 4.3)	9/227	4.0 (1.8-8.7)	1.1 (0.5 – 2.4)
	25-34	32/240	13.3 (9.8-17.8)	2.4 (1.6 – 3.7)	15/239	6.3 (4.2-9.3)	1.7 (1.0 – 2.8)
	35-54	32/476	6.7 (4.9-9.2)	1.2 (0.9 – 1.7)	19/476	4.0 (2.3-7.0)	1.1 (0.5 – 2.2)
	≥55	27/488	5.5 (3.6-8.3)	Ref	18/488	3.7 (2.2-6.2)	Ref
Usually residing in French Polynesia	Yes	207/1700	12.2 (9.9-14.8)	3.9 (1.2 -12.5)	94/1700	5.5 (4.1-7.4)	3.4 (0.5-24.9)
	No	2/64	3.1 (0.9-10.1)	Ref	1/64	1.6 (0.2-11.6)	Ref
Location	Urban	105/911	11.5 (8.4-15.6)	Ref	56/911	6.1 (4.1-9.1)	Ref
	Rural	105/859	12.2 (9.5-15.6)	1.1 (0.7 – 1.5)	41/858	4.8 (3.2-7.1)	0.8 (0.5 – 1.3)

### Bacterial skin infection

**Fig 3:**
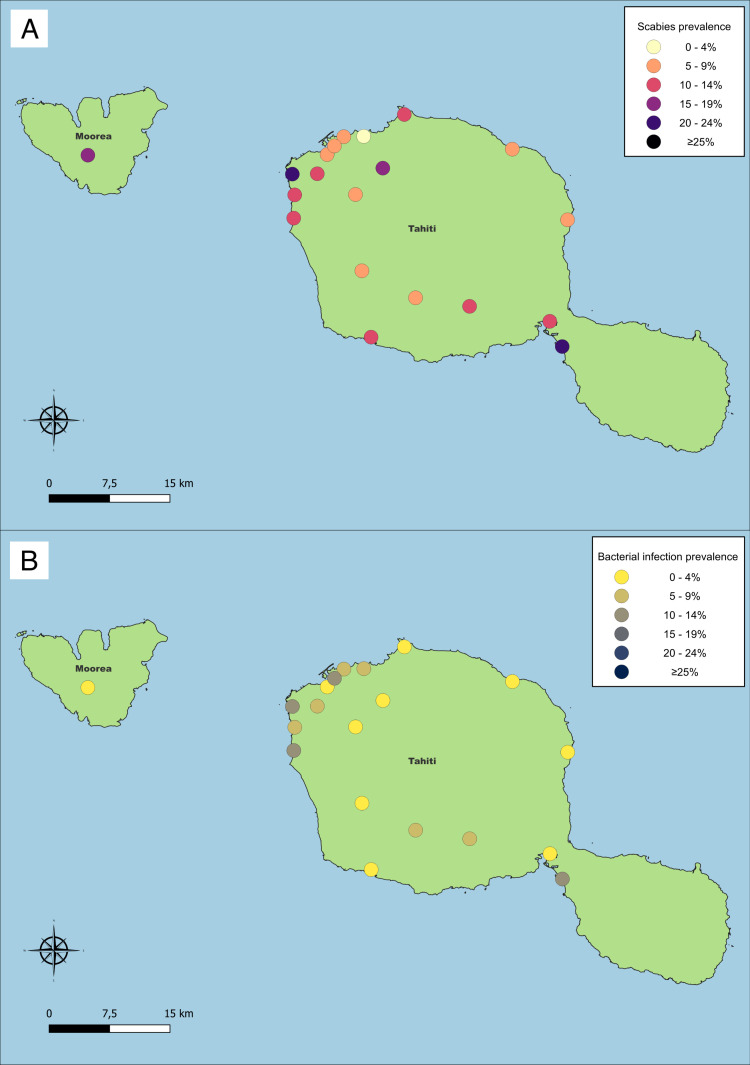
a: Geographical distribution of scabies in Tahiti and Mo’orea. The source that was used to create this map is found at data.gouv.fr. The authors understand and agree to the terms of the Creative Commons Attribution 4.0 International (CC BY 4.0) License.

We observed bacterial skin infection among 97 participants (5.5%, 95% CI 4.1–7.4%), with prevalence highest among children aged 10–14 years (Table 2 and Fig 2). Among participants with scabies, 56 (26.8%, 95% CI 20.4 – 34.4%) had bacterial skin infection, comparted to 41 among those without scabies (2.7%, RR 10.2, 95% CI 7.5–13.8). Among participants with scabies, those with at least 10 scabies lesions were more likely to have bacterial skin infection than those with fewer than 10 lesions (29 of 57, 50.8%, vs. 27 of 152 17.8%, RR 2.9, 95% CI 1.9 – 4.3, P < 0.001). The cluster-adjusted population attributable fraction of bacterial skin infection associated with scabies was 52.7% (95% CI 43.6 – 61.8%). The geographic distribution of scabies and bacterial skin infection by neighbourhood can be seen in Fig 3A and 3B.

b: Geographical distribution of bacterial skin infection in Tahiti and Mo’orea. The source that was used to create this map is found at data.gouv.fr. The authors understand and agree to the terms of the Creative Commons Attribution 4.0 International (CC BY 4.0) License.

## Discussion

Our study is the first to report the prevalence of scabies and bacterial skin infection in French Polynesia. We observed a very high prevalence of scabies (11.9%), exceeding the threshold of 10% above which treatment of the entire community with mass drug administration of ivermectin may be considered.[8,19] We also observed a moderately high prevalence of bacterial skin infection (5.5%).

We observed a strong relationship between scabies and bacterial skin infection; participants with scabies carried a ten times higher risk of bacterial skin infection than those without, and the population attributable fraction of 52.7%, indicating that if scabies was controlled the burden of bacterial skin infection in Tahiti and Mo’orea could be reduced by as much as half. We also observed that higher average household occupancy was associated with scabies and/or bacterial skin infection consistent with the epidemiological understanding of overcrowding as a driver of scabies transmission.[20] These results emphasise the need for coordinated public health strategies to combat these diseases, with a particular focus on families residing in denser living conditions.

The prevalence of scabies in our study is comparable to that observed in Solomon Islands in 2019 (scabies 15.0%, impetigo 5.6%), but less than that observed in Fiji in 2007 (scabies 23.6%, impetigo 19.6%). In all three settings, scabies prevalence was highest among young children; 27.0% among infants aged 0–1 years in Fiji, and 43.7% among school-aged children aged 5–9 years in Solomon Islands. We found that scabies prevalence in our study consistently decreased with age, which was different to the U-shaped age-specific prevalence found in Fiji and Solomon Islands.[5,6] Our study was the first to use simplified criteria for scabies diagnosis, without the requirement for presence of itch or contact history in the case definition. Such an approach has been demonstrated to be accurate in determining scabies prevalence in high prevalence, tropical countries (sensitivity 82%, specificity 98% when compared to The International Alliance for the Control of Scabies 2020 consensus criteria) and allowed for more rapid participant assessment, which was important given resource-constraints.[16]

Our study identified important considerations for planning any future survey or disease control strategies such as mass drug administration. While our initial recruitment target was 2000 participants (100 from each of 20 neighbourhoods), the rate of recruitment (44 participants per field team per day) was slower than anticipated, and we revised our recruitment target to 80 participants per neighbourhood. The main reason for slow recruitment was low household occupancy during work and school hours on weekdays; participants were recruited from less than half (48%) of the households approached, usually because no-one appeared to be home. This may have biased our study population. Other barriers to recruitment included the timing of the study relative to season (we began at the start of the wet season), living arrangements of potential participants (families living in apartments and in compounds surrounded by high opaque metal gates) and safety issues (very high rates of dog-ownership that posed risks to the field teams).

Recruitment beyond the islands of Tahiti and Mo’orea was not feasible due to the considerable resources needed to cover over 50 islands representing less than 25% of the country’s population. This may have had an impact on the generalisability of our results to the broader French Polynesian population, though this potential impact is considered small, given the movement of population between islands. There were some differences in baseline characteristics between our study and census data, including a relative under-representation of school-aged children, particularly those aged 10–14 years (4.7% vs. 7.9%), which was the cohort with the highest prevalence of bacterial skin infection, and an over-representation of adults aged ≥55, who had the lowest prevalence of both scabies and bacterial skin infection. This would suggest our findings are an underestimate of the total prevalence. It is also possible that the children recruited to our study were not representative of all children. The under-representation of children occurred despite our team purposefully conducting the first week of recruitment during school holidays. It may be that children returned to outer archipelagos during school holidays and were therefore unable to be recruited. Finally, while training and employing local nurses as skin examiners has obvious advantages in optimising the study’s cultural and linguistic acceptability, their skin assessments may have been less sensitive and less specific in detecting both scabies and impetigo compared to expert physicians, especially in cases of mild disease.[21]

Our study found a high prevalence of scabies, exceeding the 10% threshold at which community-wide disease control measures may be considered. We also found a moderate prevalence of bacterial skin infection, and determined that participants with scabies carried a ten-fold risk of bacterial skin infection compared to those without scabies. Evidence-based public health interventions are required to reduce the burden of scabies, and in doing so are likely to reduce the prevalence of bacterial skin infections and their sequelae.

## Supporting information

S1 Data (XLS)

## References

[1] WHO. Ending the neglect to attain the sustainable development goals: a road map for neglected tropical diseases 2021–2030. WHO. 2020. 196 p.

[2] Heukelbach J, Feldmeier H. Scabies. Lancet (London, England) [Internet]. 2006 May 27 [cited 2024 Feb 13];367(9524):1767–74. Available from: https://pubmed.ncbi.nlm.nih.gov/16731272/16731272 10.1016/S0140-6736(06)68772-2

[3] Scabies [Internet]. [cited 2023 Aug 20]. Available from: https://www.who.int/news-room/fact-sheets/detail/scabies

[4] Romani L, Steer AC, Whitfeld MJ, Kaldor JM. Prevalence of scabies and impetigo worldwide: a systematic review. Lancet Infect Dis. 2015;15(8):960–7. doi: 10.1016/S1473-3099(15)00132-2 26088526

[5] Romani L, Koroivueta J, Steer AC, Kama M, Kaldor JM, Wand H, et al. Scabies and impetigo prevalence and risk factors in Fiji: a national survey. PLoS Negl Trop Dis. 2015;9(3):e0003452. doi: 10.1371/journal.pntd.0003452 25738499 PMC4349858

[6] Lake SJ, Engelman D, Sokana O, Nasi T, Boara D, Grobler AC, et al. Defining the need for public health control of scabies in Solomon Islands. PLoS Negl Trop Dis. 2021;15(2):e0009142. doi: 10.1371/journal.pntd.0009142 33617544 PMC7932527

[7] Lake SJ, Kaldor JM, Hardy M, Engelman D, Steer AC, Romani L. Mass drug administration for the control of scabies: a systematic review and meta-analysis. Clin Infect Dis. 2022;75(6):959–67. doi: 10.1093/cid/ciac042 35088849 PMC9522411

[8] Engelman D, Marks M, Steer AC, Beshah A, Biswas G, Chosidow O, et al. A framework for scabies control. PLoS Negl Trop Dis. 2021;15(9):e0009661. doi: 10.1371/journal.pntd.0009661 34473725 PMC8412357

[9] WHO. Global programme to eliminate lymphatic filariasis: progress report, 2018. Vol. 94. 2019. p. 457–70.

[10] Yajima A, Ichimori K. Progress in the elimination of lymphatic filariasis in the Western Pacific Region: successes and challenges. Int Health. 2020;13(Suppl 1):S10–6. doi: 10.1093/inthealth/ihaa087 33349886 PMC7753160

[11] Nemoto T, Aubry M, Teissier Y, Paul R, Cao-Lormeau V-M, Salje H, et al. Reconstructing long-term dengue virus immunity in French Polynesia. PLoS Negl Trop Dis. 2022;16(10):e0010367. doi: 10.1371/journal.pntd.0010367 36191046 PMC9560594

[12] Musso D, Rovery C, Loukil A, Vialette V, Nguyen NL. Leprosy in French Polynesia. New Microbes New Infect. 2019;29:100514. doi: 10.1016/j.nmni.2018.10.010 30911399 PMC6416770

[13] ISPF. Institut de la statistique de la Polynésie française [Internet]. 2022 [cited 2024 Feb 17]. Available from: https://www.ispf.pf/

[14] Bartlett A, Dyer C, Kaldor J, Andersson S, Steer A, Engelman D, et al. Monitoring and Evaluation Plan for Mass Drug Administration Programs for Scabies Control. World Scabies Progr. 2021.

[15] ENA-SMART. Emergency Nutrition Assessment for Standardized Monitoring and Assessment of Relief and Transitions (ENA for SMART) Software User Manual [Internet]. 2011. p. 1–71. Available from: https://smartmethodology.org/wp-content/uploads/2014/11/ENA-Manual.pdf

[16] Tsoi SK, Lake SJ, Thean LJ, Matthews A, Sokana O, Kama M, et al. Estimation of scabies prevalence using simplified criteria and mapping procedures in three Pacific and southeast Asian countries. BMC Public Health. 2021;21(1):2060. doi: 10.1186/s12889-021-12039-2 34758806 PMC8579609

[17] Engelman D, Yoshizumi J, Hay RJ, Osti M, Micali G, Norton S, et al. The 2020 international alliance for the control of scabies consensus criteria for the diagnosis of scabies. Br J Dermatol. 2020;183(5):808–20. doi: 10.1111/bjd.18943 32034956 PMC7687112

[18] ISPF. Institut de la statistique de la Polynésie française [Internet]. 2017 [cited 2024 Jan 26]. Available from: https://data.ispf.pf/bases/Recensements/2017/Donnees_detaillees.aspx

[19] Marks M, McVernon J, McCarthy JS, Enbiale W, Hanna C, Chosidow O, et al. Diagnostics to support the control of scabies-Development of two target product profiles. PLoS Negl Trop Dis. 2022;16(8):e0010556. doi: 10.1371/journal.pntd.0010556 36040928 PMC9467343

[20] Engelman D, Cantey PT, Marks M, Solomon AW, Chang AY, Chosidow O, et al. The public health control of scabies: priorities for research and action. Lancet. 2019;394(10192):81–92. doi: 10.1016/S0140-6736(19)31136-5 31178154 PMC11257500

[21] Osti MH, Sokana O, Gorae C, Whitfeld MJ, Steer AC, Engelman D. The diagnosis of scabies by non-expert examiners: a study of diagnostic accuracy. PLoS Negl Trop Dis. 2019;13(8):e0007635. doi: 10.1371/journal.pntd.0007635 31425513 PMC6715246

